# Preparation of Glycyrrhetinic Acid Liposomes Using Lyophilization Monophase Solution Method: Preformulation, Optimization, and In Vitro Evaluation

**DOI:** 10.1186/s11671-018-2737-5

**Published:** 2018-10-16

**Authors:** Tingting Liu, Wenquan Zhu, Cuiyan Han, Xiaoyu Sui, Chang Liu, Xiaoxing Ma, Yan Dong

**Affiliations:** College of Pharmacy, Qiqihar Medical University, Qiqihar, 161006 China

**Keywords:** Glycyrrhizic acid, Liposomes, Monophase solution method, Preformulation, Cell uptake

## Abstract

In this study, glycyrrhetinic acid (GA) liposomes were successfully prepared using lyophilization monophase solution method. Preformulation studies comprised evaluation of solubility of soybean phosphatidylcholine (SPC), cholesterol, and GA in tert-butyl alcohol (TBA)/water co-solvent. The influences of TBA volume percentage on sublimation rate were investigated. GA after lyophilization using TBA/water co-solvent with different volume percentage was physicochemically characterized by DSC, XRD, and FTIR. The XRD patterns of GA show apparent amorphous nature. FTIR spectroscopy results show that no chemical structural changes occurred. Solubility studies show aqueous solubility of GA is enhanced. The optimum formulation and processing variables of 508 mg SPC, 151 mg cholesterol, 55% volume percentage of TBA, 4:1 trehalose/SPC weight ratio were obtained after investigating by means of Box-Benhnken design and selection experiment of lyoprotectant. Under the optimum conditions, satisfactory encapsulation efficiency (74.87%) and mean diameter (191 nm) of reconstituted liposomes were obtained. In vitro drug release study showed that reconstituted liposomes have sustained-release properties in two kinds of release medium. Furthermore, in vitro cell uptake study revealed that uptake process of drug-loaded liposomes by Hep G2 cells is time-dependent.

## Background

Glycyrrhetinic acid (GA), one kind of triterpene saponin, is mainly extracted from the roots of traditional Chinese medicine glycyrrhiza [1]. Studies have shown that GA has obvious antimicrobial, antiviral, and anticancer effects and it is commonly used for clinical treatments of chronic hepatitis and liver cancer [2–4]. According to the Biopharmaceutical Classification System, GA is a type II drug. Due to the low polarity, high hydrophobicity, and poor solubility of GA molecules, its oral bioavailability is relatively low [5]. Moreover, GA may cause sodium retention and potassium loss [6], which are associated with hypertension, while the adverse effects of GA seem to be dose-dependent. Therefore, using appropriate formulation strategies to increase absorption and maintain effective concentration of GA will significantly improve its bioavailability and safety.

The superiority of liposomes as drug carriers has been widely recognized [7–9]. Their functional advantages are mainly demonstrated through the following aspects: (1) liposomes have good biocompatibility and safety; (2) liposomes enhance targeted drug delivery to lymph nodes and reduce the inhibitory effects or damage that anticancer drugs have on normal cells and tissues; (3) appropriately-sized drug-carrier liposomes have enhanced permeability and retention effects at sites of solid tumors, infection, and inflammation where capillary blood vessel permeability is increased, demonstrating the ability of passive targeting; (4) liposomes can carry both hydrophobic and water-soluble drugs; and (5) the liposome surface can be modified and linked to functional groups. As a result of these advantageous characteristics, many liposome drugs have been approved.

The products obtained by conventional liposome preparation methods are aqueous liposome suspensions. However, aqueous liposome suspensions are relatively unstable and may leak, fuse, and undergo phospholipid hydrolysis during storage, resulting in limited long-term storage ability [10]. Currently, the effective way to solve these problems is to prepare proliposomes [11]. Proliposome is a powder with good fluidity that is made from dehydrated liposome components and excipients. Liposomes can be reconstructed by dispersing the proliposome in water before application. Spray-drying and freeze-drying are the two most common methods for proliposomes preparation [12], but they have several limitations in application. For example, spray-drying is not suitable for thermosensitive drugs, and can often lead to problems such as wall adherence due to the low thermal efficiency of the equipment. The structural rearrangements of the liposomal bilayers can happen during the spray-drying process [13]. The commonly used freeze-drying method is a water suspension system, but water takes a long time to be freeze-dried so this method is very costly and time-consuming.

A novel proliposomes preparation method (lyophilization monophase solution method) has been developed in recent years [14, 15]. This method involves dissolving the lipids, drug, and water-soluble lyoprotectants in a tert-butyl alcohol (TBA)/water co-solvent systems, then obtaining proliposomes by freeze-drying, following addition of water, forming a homogenous liposome suspension. This method has several advantages: (1) the addition of TBA can significantly improve the sublimation rate of ice, resulting in rapid and thorough lyophilization that is economically favorable. At the same time, rapid sublimation is beneficial for preventing lumps from collapsing [16]. (2) Lyophilization monophase solution technique is a one-step process, which is a highly effective method for large-scale liposome preparation. (3) Although not listed in the ICH Guidelines for Residual Solvents, TBA is likely to fall in the category of a class 3 low-toxicity solvent based on its similarity of LD_50_ toxicity data for other class 3 solvents [17]. (4) Sterile powder can be obtained by this method. (5) It is suitable for drugs with poor water solubility or poor water stability [18].

There have been a few reports on use of the TBA/water lyophilization system for liposome preparation. However, research on this system is inadequate and many questions still remain. For example, the variation in sublimation rate of TBA/water systems with different concentrations, changes in the solid-state property of the specific drug after lyophilization by TBA/water systems with different concentrations, and the hydration and assembly process of the lyophilized powder are still unclear. On the other hand, the solubility of a particular hydrophobic drug in TBA/water co-solvent with different proportions and temperatures is very specific. The above information is critical for the formulation and technological design of drug-carrier liposomes. Therefore, in this present study, we used the GA as a model drug to carry out preformulation investigation as described above. Furthermore, using mean diameter and entrapment efficiency as the primary evaluation measures, we optimized the formulation and processing variables of GA-liposomes prepared by lyophilization monophase solution method using Box-Benhnken design. The effects of the lyophilization protectant types on the quality of liposomes were evaluated, as were the in vitro release of liposomes and their uptake by hepatoma cells.

## Methods/Experimental

### Materials

Glycyrrhetinic acid (> 98% pure) was obtained from Dalian Meilun Biology Technology Co., Ltd. (Dalian, China). Soybean phosphatidylcholine (Lipoid S100) was purchased from Lipoid GmbH (Ludwigshafen, Germany). Cholesterol was purchased from J&K Scientific Ltd. (Beijing, China). Reference compound of GA was purchased from National Institutes for Food and Drug Control (Beijing, China). FITC-PEG-DSPE (molecular weight 2000) was purchased from Shanghai Ponsure Biotech, Inc. (Shanghai, China). Tert-butyl alcohol (> 98%) and all other reagents, if not otherwise specified, were purchased from Sinopharm Chemical Reagent Co., Ltd. (Beijing, China). Deionized water was prepared by a Milli-Q water purification system (Millipore, Bedford, MA, USA).

### Solubility Study of GA, SPC, and Cholesterol in TBA/Water Co-solvent System

The saturated TBA-water solutions (30 ml) of GA with different TBA volume percentage were prepared by stirring an excess of drug in the corresponding vehicle at 25 °C, 30 °C, 35 °C, 40 °C, and 45 °C for 72 h. After centrifugation (15 min at 3000 rpm), the supernatant was passed through a 0.45 μm microporous filters. The saturation solubility of GA was measured by HPLC after adequate dilution. Three replicates were performed in each TBA/water co-solvent. HPLC analysis was performed on an LabAlliance (model Series III) HPLC system (Lab Alliance, Tianjin, China) equipped with a quaternary pump, an autosampler, and a column compartment, coupled to a UV detector. Separation was performed on a C18 column (4.6 mm × 250 mm; 5 μm; Dikma Technologies, Beijing, China); methanol and water (90:10 *V*/*V*) were used as mobile phase at a flow rate of 1.0 ml/min. The analytes were detected by UV detector at 250 nm.

Solubility of soybean phosphatidylcholine (SPC) (or cholesterol) in TBA/water co-solvent system was estimated using turbidimetric method [19, 20]. Briefly, 10 mg SPC (or cholesterol) was dissolved in TBA at 25 °C, 30 °C, 35 °C, 40 °C, and 45 °C to obtain a clear solution; the temperature was maintained throughout the duration of experiment. An increasing amount of purified water at the same temperature was added to TBA solution of SPC (or cholesterol) at 25 °C until the turbidity first took place, and critical water volume value was recorded. Turbidity can be identified by detecting the absorption value at 655 nm (> 0.04) against the blank solution (purified water) on a T6 model UV-Vis spectrophotometer (Purkinje General Instrument Co., Ltd., Beijing).

### Preparation of Liposomes Using Lyophilization Monophase Solution Method

GA, SPC, and cholesterol was dissolved in TBA at 45 °C, and water-soluble lyoprotectant such as mannitol, lactose, sucrose, and trehalose was dissolved in 45 °C water. Then these two solutions were mixed in appropriate ratios to get a third clear isotropic monophase solution (total volume 60 ml). After the monophase solution was sterilized by filtration through 0.22 μm pores, it was filled into the 10 ml freeze-drying vials with a fill volume of 2.0 ml. After prefreezing for 12 h at − 40 °C, freeze-drying was carried out at a shelf temperature of − 50 °C for 24 h with a chamber pressure of 1–20 Pa in a lyophilizer (SJIA-10N, Ningbo Shuangjia Science Technology Development Co., Ltd., China).

### Measurement of Particle Size and Encapsulation Efficiency of Liposomes

The liposomes suspension was prepared by adding 5 mg proliposomes powder to 5 ml purified water and subsequent vortex agitation for 1 min twice with a 15-min interval for complete hydration. The size analysis of the liposomes was characterized by using laser particle size analyzer (Nano ZS90 Malvern Instruments, UK).

The encapsulation efficiency of GA in liposomes was determined by the ultrafiltration-centrifugation technique. Briefly, pipette 1 ml of liposomal dispersion (500 μg proliposomes in 5 ml purified water) into a 10 ml volumetric flask, followed by adding 5 ml of purified water, 2 ml of acetone, and dilute to 10 ml with purified water. Transfer 0.5 ml of this suspension into the upper chamber of the centrifuge filter (Amicon Ultra-0.5, Millipore, Cdduounty Cork, Ireland) with molecular weight cut off of 50 kDa, which was centrifuged at 10,000 rpm for 30 min at 15 °C using an ultracentrifuge (CP70MX, Hitachi Koki Co., Ltd., Japan). Then, 20 μl ultrafiltrate was injected into HPLC system at a UV absorption wavelength of 250 nm, and the content of GA was called the content of free drug. The encapsulation efficiency (EE) was calculated according to the following equations

1 $$ \mathrm{EE}\left(\%\right)=\frac{W_{\mathrm{total}}-{W}_{\mathrm{free}}}{W_{\mathrm{total}}}\times 100 $$

where *W*_free_ is the amount of free drug and *W*_total_ is the amount of total drug.

### Determination of the Sublimation Rate of TBA/Water Mixtures

One milliliter of TBA/water mixtures of different TBA volume percentage (10%, 20%, 30%, 40%, 50%, 60%, 70%, 80%, and 90%) were put into 10 ml freeze-drying vials, respectively. The TBA/water mixtures were pre-frozen at − 40 °C for 12 h and then lyophilized by lyophilizer (SJIA-10N, Ningbo Shuangjia Science Technology Development Co., Ltd., China) at − 50 °C. Time was recorded when TBA/water mixtures completely disappear from freeze-drying vials, and the sublimation rate was calculated by dividing volume (μl) by the time (min).

### Determination of Saturated Vapor Pressure of TBA/Water Mixtures

The details of the experimental apparatus and the operation procedure were described elsewhere [21, 22]. The vapor pressures of the system TBA/water (10%, 20%, 30%, 40%, 50%, 60%, 70%, 80%, and 90%) were measured by a static method. The apparatus was composed of a working ebulliometer filled with TBA/water mixture, a reference ebulliometer filled with pure water, a buffer vessel, two condensers, two temperature measurement, and a pressure control system. The equilibrium pressure of the system was determined by the boiling temperature of pure water in the reference ebulliometer in terms of the temperature–pressure relation represented by Antoine equation [23].

### Determination of GA Solubility

The solubility in water of free GA was determined by adding excess GA (10 mg) to 10 ml of pure water under magnetic stirring (300 rpm) in a thermostatically controlled water bath (DF-101S, Henan Yuhua instrument Co., Ltd., China) at 25 °C until equilibrium was achieved (48 h). The samples were filtered through a 0.45 μm membrane filter, suitably diluted with methanol, and analyzed by HPLC [24]. Experiments were performed in triplicate.

### Surface Morphology Observation of Pre-frozen TBA/Water Mixtures

Five milliliters of water/tert-butanol mixtures were poured into a 90-mm Petri Dish, then was frozen out in the cold-trap (− 40 °C); the frozen samples were observed using an XSP-4C optical microscope (Shanghai Changfang Optical Instrument Co. Ltd., Shanghai, China).

### Transmission Electron Microscopy

Liposomes appearance was observed by Hitachi HT7700 transmission electron microscopy (TEM) (Hitachi, Japan) at an accelerating voltage of 100 kV. The liposomes suspension was obtained by adding 5 mg proliposomes powder to 5 ml purified water at room temperature, mixed by vortex for 10 s, and then was left standing for 30 s. A drop was withdrawn with a micropipette then placed on a carbon-coated copper grid. The excess of the suspension was removed by blotting the grid with a filter paper. Negative staining using a 1% phosphotungstic acid solution (*w*/*w*, pH 7.1) was directly made on the deposit. The excess was removed with a filter paper and deposit was left to dry before analysis.

### Fourier Transform Infrared Spectroscopy

The Fourier transform infrared spectroscopy (FTIR) spectra of samples were obtained on a Nicolet 6700 FTIR spectrophotometer (Thermo Scientific, Waltham, MA, USA). Every sample and potassium bromide was mixed by an agate mortar and compressed into a thin disc. The scanning range was 4000–400 cm^−1^ and the resolution was 4 cm^−1^.

### Differential Scanning Calorimetry

Differential scanning calorimetry (DSC) measurements were performed on a HSC-1 DSC scanning calorimeter (Hengjiu Instrument, Ltd., Beijing, China). Samples of 15 mg were placed in aluminum pans and sealed in the sample pan press. The probes were heated from 25 to 350 °C at a rate of 10 °C/min under nitrogen atmosphere.

### X-ray Diffraction

The structural properties of samples were obtained using the D8 Focus X-ray diffractometer (Bruker, Germany) with Cu-Kα radiation. Measurements were performed at a voltage of 40 kV and 40 mA. Samples were scanned from 5° to 60°, and the scanned rate was 5°/min.

### Stability of GA Proliposome

GA proliposome powders were transferred into a glass bottle, filled with nitrogen, sealed, and stored away from light at the room temperature. The stability testing was carried out for 6 months by using entrapment efficiency and particle size of the reconstituted liposomes as the indexes.

### In Vitro Drug Release

Release of GA from liposomes was observed using the dialysis method at 37 ± 0.5 °C. After reconstituting liposomes in PBS (pH 7.4) or normal saline to make 0.5 mg/ml of GA, an aliquot of each liposomal dispersion (5 ml) was placed in a dialysis bag (molecular weight cut-off 8000–14,000 Da) and was tightly sealed. Then, the tube was immersed in 150 ml of release medium, PBS (pH 7.4), or normal saline containing 0.1% (*v*/*v*) Tween 80 to maintain sink condition [25, 26]. While stirring the release medium using the magnetic stirrer at 300 rpm, samples (1.5 ml) were taken at predetermined time intervals from the release medium for 12 h, which was refilled with the same volume of fresh medium. Concentration of GA was determined by HPLC after appropriate dilution with methanol.

### In Vitro Cellular Uptake

The fluorescence liposomes were prepared by lyophilization monophase solution method. Briefly, a mixture of 30 mg GA, 254 mg SPC, 75.5 mg cholesterol, and 21.2 mg FITC-PEG-DSPE were dissolved in TBA. Further, 1016 mg trehalose was dissolved in water. Then these two solutions were mixed to get a clear monophase solution (total volume 30 ml). After the monophase solution was sterilized by filtration through 0.22 μm pores, it was filled into the 10-ml freeze-drying vials with a fill volume of 2.0 ml, then lyophilized for 24 h and added water to reconstitute liposomes until use.

The HepG2 cells (Wanleibio, Co., Ltd., Shenyang, China) were cultured in DMEM with 10% FBS (fetal bovine serum). The cells were plated until 90% confluence was achieved in 6-well plates, and the cells were cultured in a humidified incubator at 37.0 °C with 5.0% CO_2_. After 24-h incubation, 200 μl FITC-GA-liposomes suspension were added to 1 ml of the HepG2 cells suspension (1 × 10^4^ cells per well). Following incubation for 0.5 h, 1 h, 2 h, and 4 h, cells were washed three times with pH 7.4 PBS, and extracellular fluorescence was quenched with a 0.4% (*w*/*v*) Trypan blue solution. Cells were lysed with 1% (*w*/*v*) Triton X100. The fluorescence intensity of the cellular lysate at 495 nm excitation and 520 nm emission was measured using a RF5301 fluorescence spectrophotometer (Shimadzu, Tokyo, Japan). Relative fluorescence values were converted to phospholipid concentrations based on a standard curve of phospholipid concentration versus FITC fluorescence intensity measured in the cell lysis buffer. Protein concentration was determined using BCA protein assay kit (Pierce, Rockford, IL, USA). Uptake was expressed as the amount of phospholipids versus per milligrams cellular protein [27].

## Results and Discussion

### Preformulation Study

#### Solubility Study

Since liposomes are prepared using lyophilization monophase solution method, a solubility study was performed to ensure that the drug and carrier material could dissolve in the TBA/water solution prior to lyophilization.

**Fig. 1:**
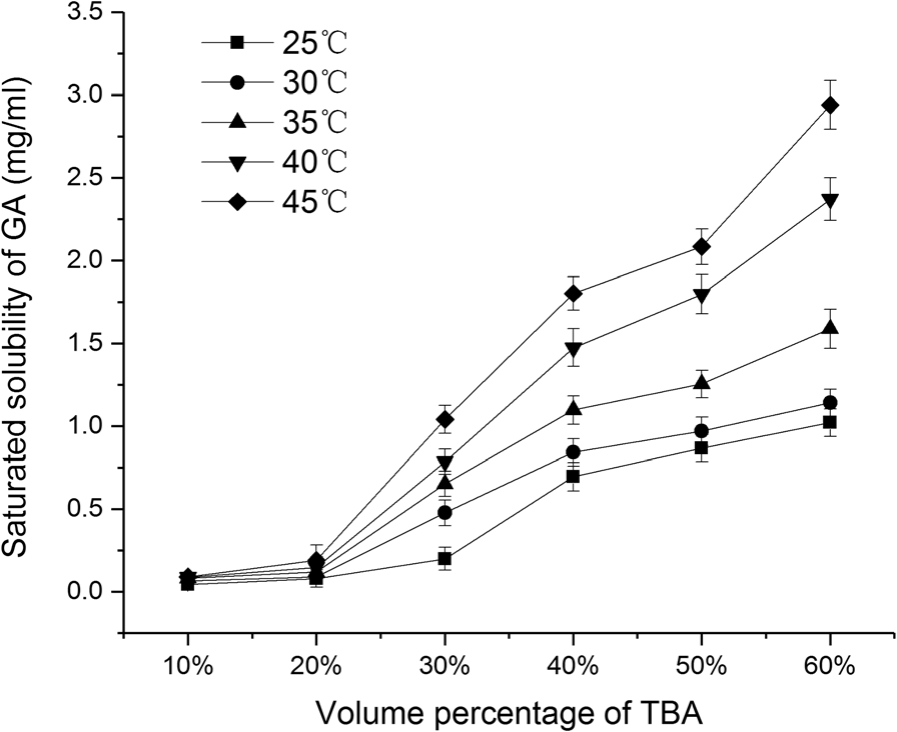
Saturated solubility of GA in TBA/water co-solvent with different volume percentage (mean ± SD, *n* = 3)

**Fig. 2:**
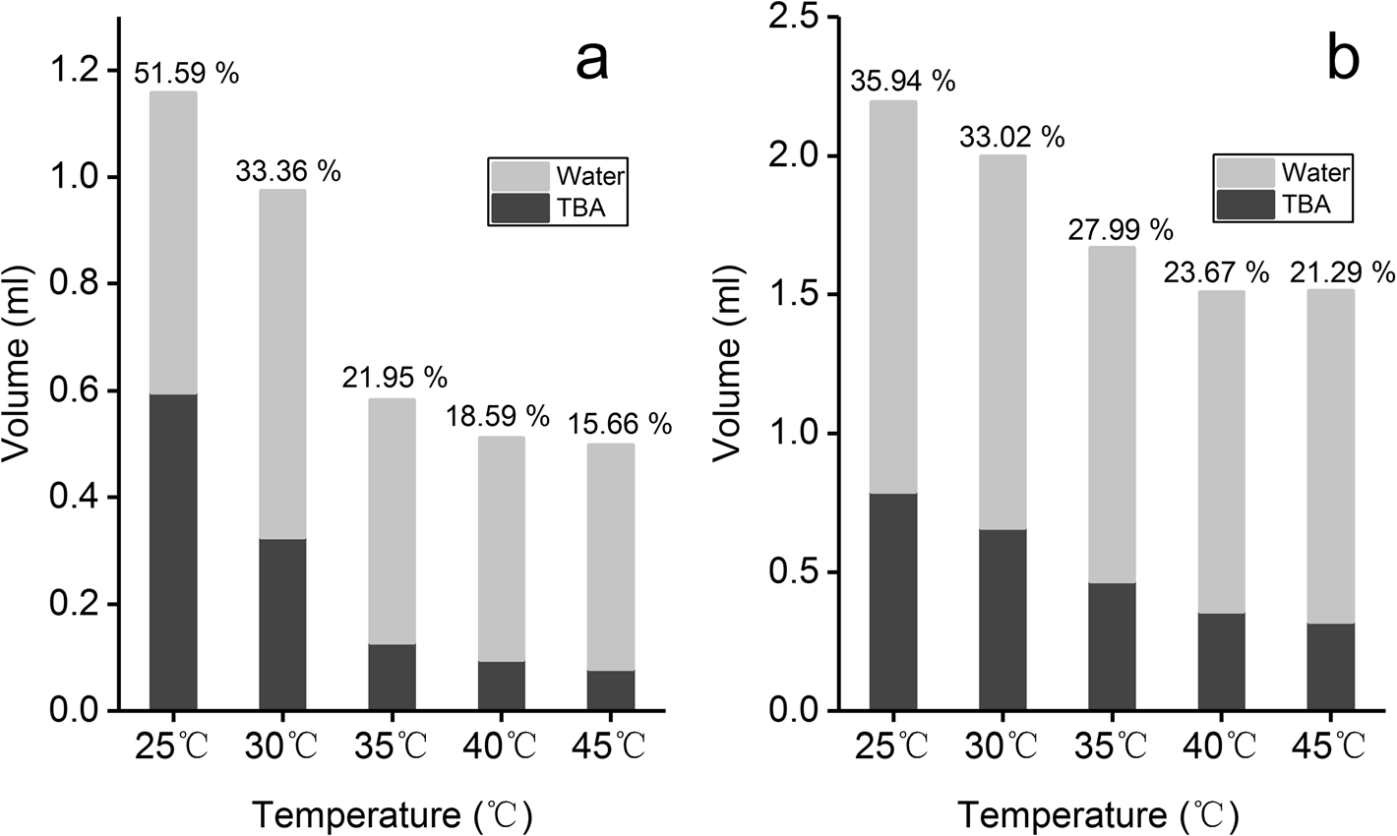
Solubility of SPC (**a**) and cholesterol (**b**) in TBA/water co-solvent with different volume percentage

Figure 1 shows the changes in the saturated solubility of GA in TBA/water co-solvent system with different volume percentage. Within 25 °C to 45 °C, the saturated solubility of GA continuously increased with increasing TBA volume percentage from 10 to 60%, and the saturated solubility of GA was > 0.5 mg/ml when TBA volume percentage was > 40%. On the other hand, the saturated solubility of GA increased with increasing temperature of the TBA/water solution, when maintained at the same volume percentage. The difference in solubility under different temperatures became increasingly apparent when the volume percentage of TBA reached 30%. The solubility of soybean phospholipids and cholesterol in the TBA/water co-solvent system is shown in the stacked column graph (Fig. 2). Figure 2a, b represents the volume of TBA/water mixture needed for a unit (1 mg) of phospholipids and cholesterol to reach saturated solubility under different temperatures, respectively. The gray area represents the volume of water and the black area represents the volume of TBA. As the temperature increased gradually from 25 to 45 °C, the total volume of TBA/water co-solvent and the volume percentage of TBA (labels on the columns) needed to dissolve 1 mg of phospholipid decreased gradually (Fig. 2a). As the temperature increased beyond 35 °C, the volume of TBA needed reduced significantly and was below 0.15 ml. Similarly, as the temperature gradually increased from 25 to 45 °C, there was a reduction in the TBA volume needed to dissolve 1 mg of cholesterol, whereas the TBA volume percentage showed a trend toward a gradually decrease. The above results demonstrated that temperature and TBA volume percentage greatly affect the solubility of phospholipids, cholesterol, and GA.

#### Comparison of the Sublimation Rate of the TBA/Water Co-solvent System with Different Volume Percentage

**Fig. 3:**
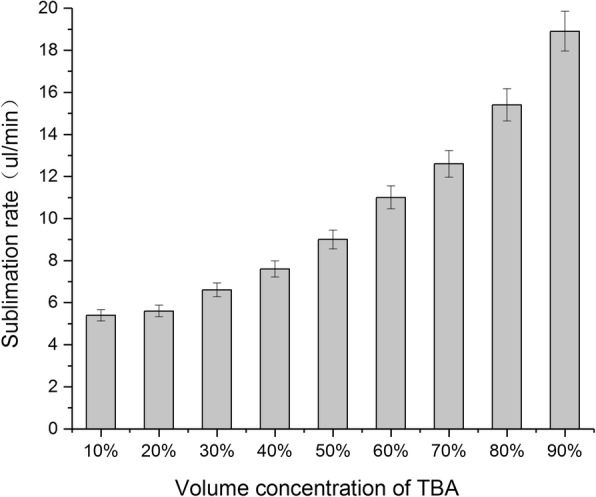
Sublimation rate of TBA/water co-solvent with different volume percentage (mean ± SD, *n* = 3)

Sublimation rate directly affects the production efficiency of lyophilized powder. A faster sublimation rate is more economical and can prevent collapse of materials [16]. In this study, we examined the sublimation rates of different concentrations of TBA/water systems. As shown in Fig. 3, the sublimation rate of the mixed solvent gradually increased as the volume percentage of TBA increased from 10 to 90%. Furthermore, the sublimation rate reached above 10 μl/min as the volume percentage exceeded 60%.

**Fig. 4:**
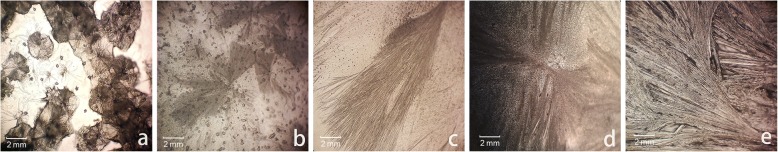
Surface morphology of TBA/water co-solvent of different TBA volume percentage by optical microscope (× 100 magnification). **a** 40%. **b** 50%. **c** 60%. **d** 70%. **e** 80%

In order to identify the reason for the difference in sublimation rate of TBA with different volume percentage, we first examined the surface morphology of the frozen samples. Figure 4 contains the optical microscopic images of the TBA/water solution with 40% to 80% volume percentage (TBA with < 30% volume percentage could not be examined as it rapidly melted under the optical microscope). Compared with TBA with 40% volume percentage, TBA with > 50% volume has clear and scattered needle-shaped structures. We speculate that as the volume percentage of TBA increases, the diameter of the needle-shaped crystals becomes smaller, resulting in an increase in the specific surface area and therefore an increase in sublimation rate.

**Fig. 5:**
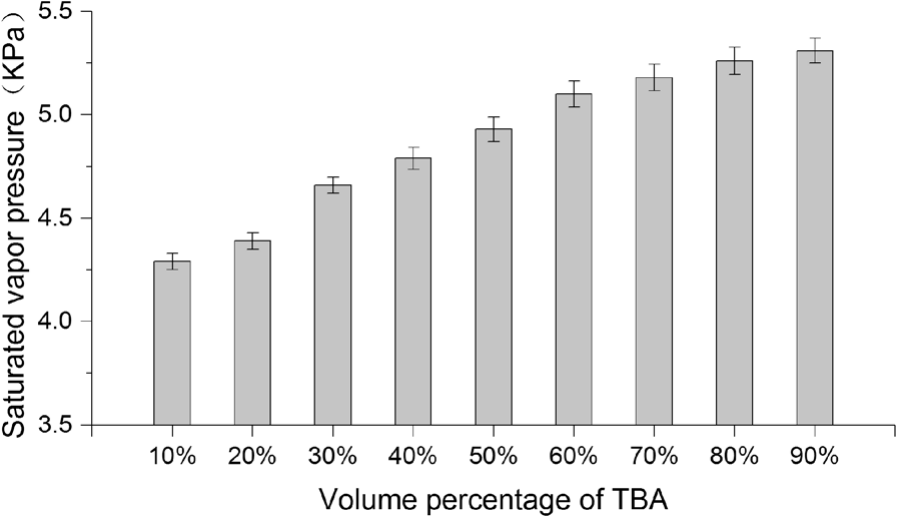
Saturated vapor pressure of TBA/water co-solvent with different volume percentage (mean ± SD, *n* = 3)

In addition, we also measured the saturated vapor pressure of the TBA/water co-solvent system with different volume percentage at 25 °C. Figure 5 is a bar graph of the changes in saturated vapor pressure of TBA/water co-solvent system with different TBA volume percentage. As shown in the figure, the saturated vapor pressure of the mixed solvent tends to gradually increase as the volume percentage of TBA increases. As temperature is positively correlated with saturated vapor pressure, as per the Antoine equation (Eq. 2), we can deduce that the saturated vapor pressure of the co-solvent system under lyophilization temperature (− 50 °C) will increase as the volume percentage of TBA increases, and this may be one of the reasons for the gradual increase in sublimation rate.

2 $$ {\log}_{10}p=A-\frac{B}{T} $$

where *p* is the vapor pressure, *T* is temperature, *A* and *B* are component-specific constants.

#### Effect of the Lyophilization on the Physical and Chemical Properties of GA in the TBA/Water Co-solvent System with Different Volume Percentage

In order to investigate the effect of lyophilization on physicochemical properties of GA in TBA/water co-solvent system, the following experiment was performed. Ten milligrams of GA was dissolved in 8 ml TBA/water co-solvent of different TBA volume percentages (40%, 50%, 60%, 70%, and 80%). After the monophase solution was sterilized by filtration through 0.22 μm pores, it was filled into the 10 ml freeze-drying vials with a fill volume of 2.0 ml. Freeze-drying was carried out at − 50 °C for 24 h by lyophilizer.

**Fig. 6:**
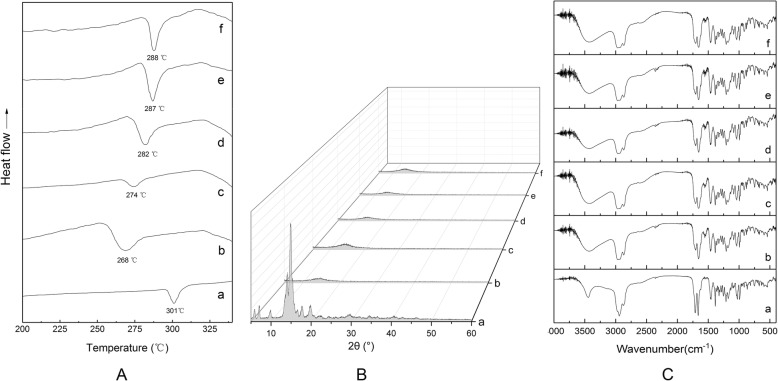
DSC (**a**), XRD (**b**), and FTIR (**c**) of GA after lyophilization in TBA/water co-solvent with different volume percentage; (a) GA, (b) 40% TBA, (**c**) 50% TBA, (d) 60% TBA, (e) 70% TBA, and (f) 80% TBA

The DSC spectra of the lyophilized powder after dissolving GA in TBA/water co-solvent system with different TBA volume percentage is shown in Fig. 6a. The DSC curve of the raw drug shows an obvious endothermic peak at 301 °C, which is the melting point of GA. Lyophilization in TBA/water co-solvent system with different TBA volume percentage caused a forward shift of the GA melting peak. The magnitude of the melting peak shift increased as TBA volume percentage decreased.

Previous research has already shown that the concentration of TBA can deeply affect formation of a complex mixture of crystalline, amorphous, or metastable phases [28]. In some cases, the use of TBA can result in reduction in crystallinity, and another case is the opposite [29].

The X-ray diffraction (XRD) spectra of the lyophilized powder after dissolving GA in TBA/water co-solvent system with different TBA volume percentage are shown in Fig. 6b. The XRD spectrum of the raw drug shows several distinct crystal diffraction peaks between 5° and 20°. Lyophilization in TBA/water co-solvent system with different TBA volume percentage caused the disappearance of diffraction peaks at 5° to 20° in the XRD spectra of the samples. This indicated that the original drug crystal had become amorphous.

The FTIR spectra of the lyophilized powder after dissolving GA in TBA/water co-solvent system with different TBA volume percentage are shown in Fig. 6c. The shape of the FTIR spectrum of the raw drug is consistent with those of the lyophilized powder in TBA/water co-solvent system with different TBA volume percentage within the 4000–400 cm^−1^ range. There was no emergence of characteristic peaks for new functional groups, demonstrating that the chemical structure of GA remained the same after lyophilization in TBA with different volume percentage.

**Fig. 7:**
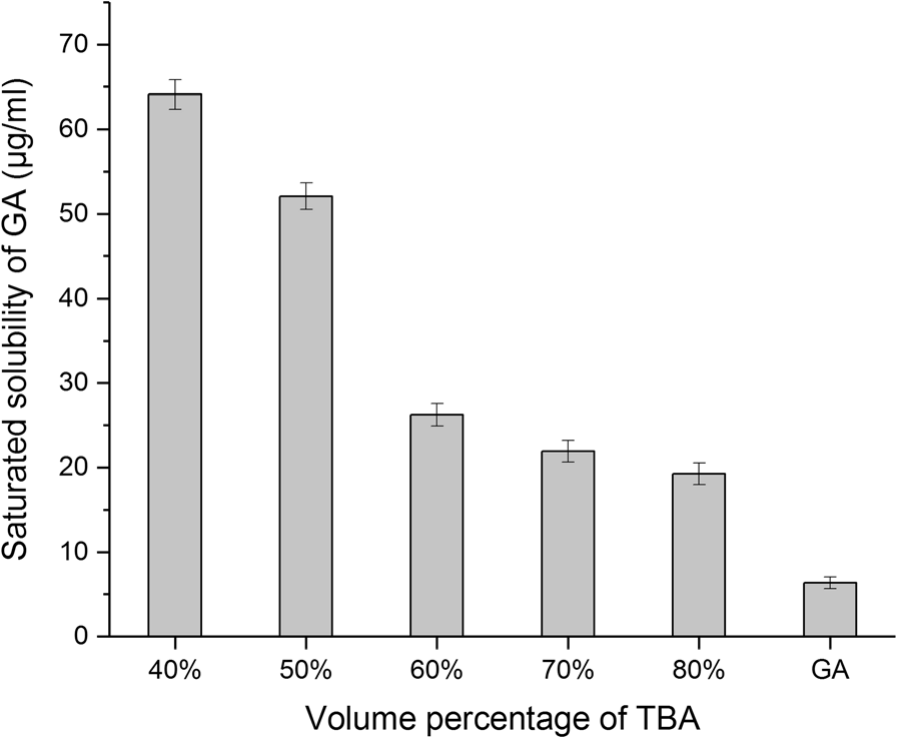
Aqueous solubility of GA lyophilization in TBA/water co-solvent with different volume percentage (mean ± SD, *n* = 3)

The change from a crystalline to an amorphous form can alter drug solubility thereby affecting proliposome encapsulation during hydrated reconstruction. In this study, we measured the aqueous solubility of lyophilized GA powder at 25 °C. We found that the saturated solubility of the lyophilized GA in water decreased gradually from 64.10 to 19.27 μg/ml as TBA volume percentage increased from 40 to 80%. However, it was still significantly higher than the solubility in water of the raw drug (6.36 μg/ml) indicating that the change from a crystalline to an amorphous structure during lyophilization does affect solubility of the raw drug (Fig. 7).

### Single-Factor Experiment

There are many factors that can influence the quality of liposomes. It is well known that phospholipid/drug ratios have an effect on encapsulate quality of the drug [30]. Moderate amounts of cholesterol can increase the ordered arrangement of lipid membrane and stability. However, high content of cholesterol in the liposome can decrease the flexibility of membrane and thereby hinder the penetration of drug into the lipid bilayer [31]. In this study, we selected three factors that impact on liposome quality and performed a single-factor study to determine the appropriate values for subsequent optimization tests, including quantity of SPC, quantity of cholesterol, and volume percentage of TBA in the co-solvent. The quality of liposomes was evaluated in terms of encapsulation efficiency and mean diameter. Each experiment was performed in triplicate with all other parameters set to constant value, GA 60 mg, pre-freeze temperature − 40 °C, pre-freeze time 12 h. In this study, we compared the results via a scoring system, giving equal weight to both encapsulation rate and mean diameter. Scoring was conducted as follows:

3 $$ \mathrm{Score}=\frac{\mathrm{EE}}{\mathrm{MEE}}\times 50\%-\frac{\mathrm{MD}}{\mathrm{MMD}}\times 50\% $$

where EE is encapsulation efficiency, MEE is maximum encapsulation efficiency of the group, MD is mean diameter, and MMD is maximum mean diameter of the group.

The experimental design and result are shown in Table 1. As can be seen in the table, within the range tested in this experiment, the highest score can be obtained separately when the amount of SPC is 480 mg (drug-SPC ratio of 1:8, *w*/*w*), the amount of cholesterol is120 mg (cholesterol-SPC ratio of 1:4, *w*/*w*), and volume percentage of TBA in the co-solvent is 50%. Therefore, these parameters were chosen as the center level of response surface optimization design, respectively.

**Table 1:** Single-factor experiments

Factor		MD (nm)^a^	EE (%)^b^	Score	Other condition
Quantity of SPC (mg)	240	221.8	45.63	− 0.052	Quantity of cholesterol 60 mg; volume percentage of TBA 50%
	360	224.6	55.86	0.019	
	480	230.4	64.47	0.073	
	600	245.8	66.49	0.061	
	720	284.6	64.77	− 0.020	
Quantity of cholesterol (mg)	60	225.3	56.35	0.015	Quantity of SPC 480 mg; volume percentage of TBA 50%
	120	230.4	64.47	0.069	
	180	238.6	63.13	0.043	
	240	235.8	60.57	0.029	
	300	267.2	55.62	− 0.069	
Volume percentage of TBA (%)	40%	230.4	64.47	0.056	Quantity of SPC 480 mg; quantity of cholesterol 120 mg
	50%	226.7	65.13	0.068	
	60%	219.4	61.83	0.056	
	70%	224.2	62.72	0.054	

^a^*MD* mean diameter

^b^*EE* entrapment efficiency

### Parameter Optimization by Box-Benhnken Design

To further study the interactions between the various factors, parameter optimization was performed by Box-Benhnken design. Based on the results of single-factor experiments, we investigated and optimized the interactions between the parameters, including quantity of SPC (*X*_1_), quantity of cholesterol (*X*_2_), volume percentage of TBA (*X*_3_) by Box-Benhnken design (BBD). Encapsulation efficiency (*Y*_1_) and mean diameter (*Y*_2_) were selected as responses. Optimization process was undertaken with desirability function to optimize the two responses simultaneously. We suppose that *Y*_1_ and *Y*_2_ have the same weightiness (importance). *Y*_1_ had to be maximized, while *Y*_2_ had to be minimized. The desirable ranges are from 0 to 1 (least to most desirable). Experimental design and results are shown in Table 2. To find the most important effects and interactions, analysis of variance (ANOVA) was calculated by statistical software, Design Expert trial version 8.03 (Stat-Ease, Inc., Minneapolis, USA). Two quadratic models were selected as suitable statistical model for optimization for two responses encapsulation efficiency and mean diameter. The results of ANOVA relating encapsulation efficiency as response were shown in Table 3, indicating that the model was significant for all factors investigated with *F* value of 12.81 (*P* < 0.05). In this case, *X*_1_, *X*_2_, *X*_1_*X*_2_, *X*_1_*X*_1_, *X*_2_*X*_2_ were significant model terms (*P* < 0.05), demonstrating that the influences of the factors (*X*_1_ and *X*_2_) on encapsulation efficiency were not simply linear. The interaction terms were notably significant, indicating good interactions between the factors. On the contrary, the ANOVA results relating mean diameter as response (Table 3) indicated that the model was not significant for all factors investigated with *F* value of 1.9 (*P* > 0.05). In this case, *X*_3_ were significant model terms (*P* < 0.05), demonstrating that volume percentage of TBA have significant influence on mean diameter, while quantity of SPC (*X*_1_) and quantity of cholesterol (*X*_2_) do not have a significant effect (*P* > 0.05). Moreover, there were no significant interactions between the three variables.

**Table 2:** Parameter optimization by response surface methodology

No.	Factor^a^			Dependent variables^b^	
	*X*_1_	*X*_2_	*X*_3_	MD (nm)	EE (%)
1	360	180	50	224.7	56.26
2	480	120	50	217.9	67.53
3	480	180	40	239.5	63.82
4	600	60	50	229.3	60.24
5	600	180	50	226.8	68.61
6	480	120	50	220.3	70.42
7	480	60	60	218.6	61.47
8	480	120	50	228.7	66.25
9	360	120	40	233.3	58.35
10	480	120	50	216.4	67.52
11	360	120	60	223.3	57.26
12	480	180	60	215.7	67.25
13	480	60	40	233.2	63.86
14	600	120	40	230.8	67.25
15	360	60	50	218.9	57.82
16	600	120	60	234.6	67.74
17	480	120	50	225.1	71.28

^a^*X*_1_ quantity of SPC, *X*_2_ quantity of cholesterol, and *X*_3_ volume percentage of TBA

^b^*MD* mean diameter, *EE* entrapment efficiency

**Table 3:** Analysis of variance (ANOVA) for response quadratic surface model

Index^a^	Source^b^	Sum of squares	Mean square	*F* value	*P* value^c^
EE	Model	353.64	39.29	12.81	0.0014
	*X*_1_	145.78	145.78	47.54	0.0002
	*X*_2_	19.69	19.69	6.42	0.0390
	*X*_3_	0.024	0.024	7.89E-03	0.9317
	*X*_1_ *X*_2_	24.65	24.65	8.04	0.0252
	*X*_1_ *X*_3_	0.62	0.62	0.20	0.6655
	*X*_2_ *X*_3_	8.47	8.47	2.76	0.1405
	*X*_1_ *X*_1_	91.39	91.39	29.80	0.0009
	*X*_2_ *X*_2_	43.35	43.35	14.14	0.0071
	*X*_3_ *X*_3_	7.02	7.02	2.29	0.1740
MD	Model	576.73	64.08	1.90	0.2052
	*X*_1_	56.71	56.71	1.68	0.2361
	*X*_2_	5.61	5.61	0.17	0.6957
	*X*_3_	248.65	248.65	7.36	0.0300
	*X*_1_ *X*_2_	17.22	17.22	0.51	0.4982
	*X*_1_ *X*_3_	47.61	47.61	1.41	0.2738
	*X*_2_ *X*_3_	21.16	21.16	0.63	0.4546
	*X*_1_ *X*_1_	51.51	51.51	1.53	0.2567
	*X*_2_ *X*_2_	0.27	0.27	7.95E-03	0.9314
	*X*_3_ *X*_3_	119.28	119.28	3.53	0.1022

^a^*MD* mean diameter, *EE* entrapment efficiency

^b^*X*_1_ quantity of SPC, *X*_2_ quantity of cholesterol, *X*_3_ volume percentage of TBA

^c^*P* value less than 0.05 were considered statistically significant

**Fig. 8:**
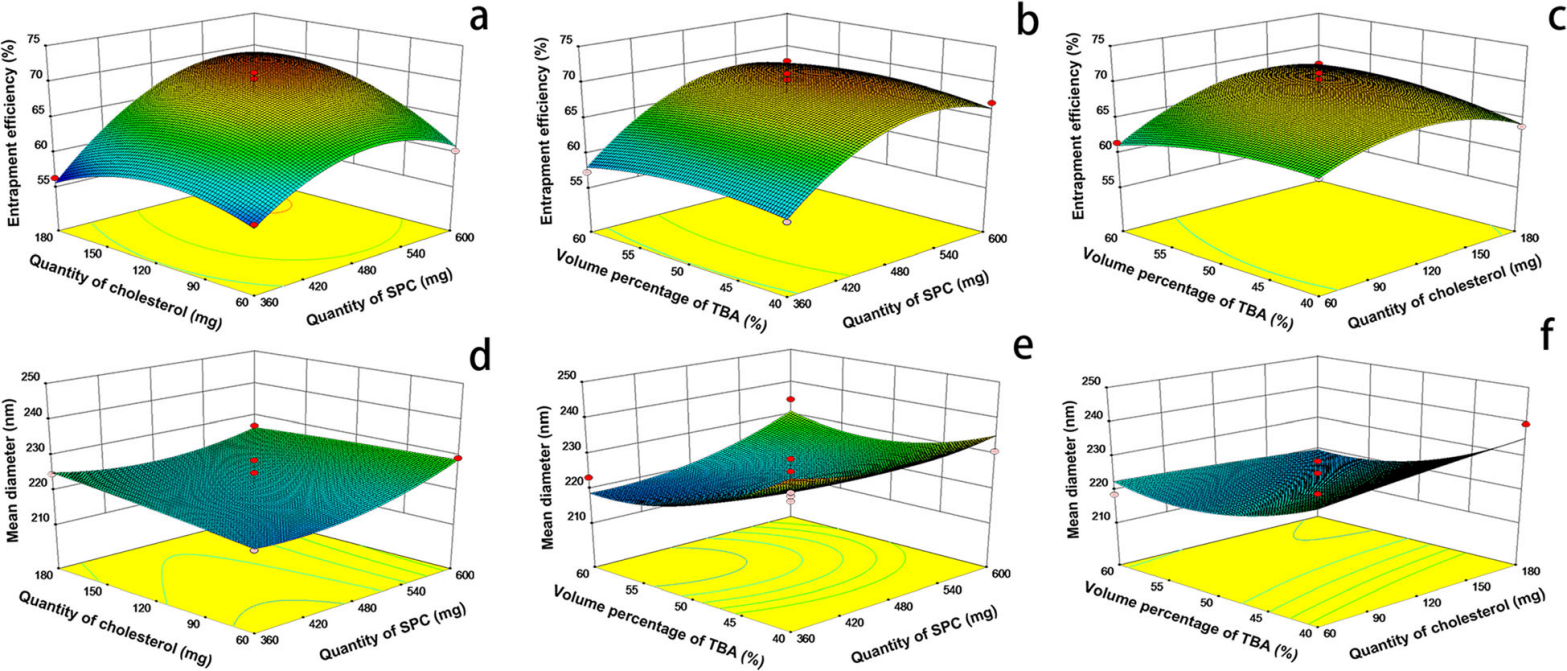
Three dimensional plots of the effect of X X (**a**), X X (**b**) and X X (**c**) on encapsulation efficiency and the effect of X X (d), X X (e) and X X (f) on mean diameter

In order to provide a better visualization of the effect of the independent variables on the two responses and desirability value, three-dimensional profiles of multiple non-linear regression models are depicted in Fig. 8. Figure 8a–f presented the interaction of *X*_1_, *X*_2_, and *X*_3_ under encapsulation efficiency and mean diameter as response respectively. The three-dimensional profiles demonstrated how three pairs of parameters affect the encapsulation efficiency and mean diameter of reconstituted liposomes. For encapsulation efficiency, all the three surfaces are upper convex (Fig. 8a–c), with a maximum point in the center of the experimental domain, which demonstrated that there are good interactions between the three variables. For mean diameter, the shape of Fig. 8d is similar to flat surface, indicating that *X*_1_ and *X*_2_ have less effect on mean diameter. The surface contours of Fig. 8e, f both showed a slope; the mean diameter was decreased by increasing the volume percentage of TBA, indicating that factor *X*_3_ had an obvious effect on mean diameter but there was no obvious interaction between *X*_3_ and the other two factors.

Based on the quadratic model, the optimal conditions for liposomes preparation calculated by software were as follows: 508 mg phospholipid quantity, 151 mg cholesterol quantity, and 55% volume percentage of TBA. Under these conditions, the encapsulation efficiency and mean diameter were found to be 68.55% and 220 nm, respectively.

### Selection of the Type and Dosage of Lyoprotectant

**Fig. 9:**
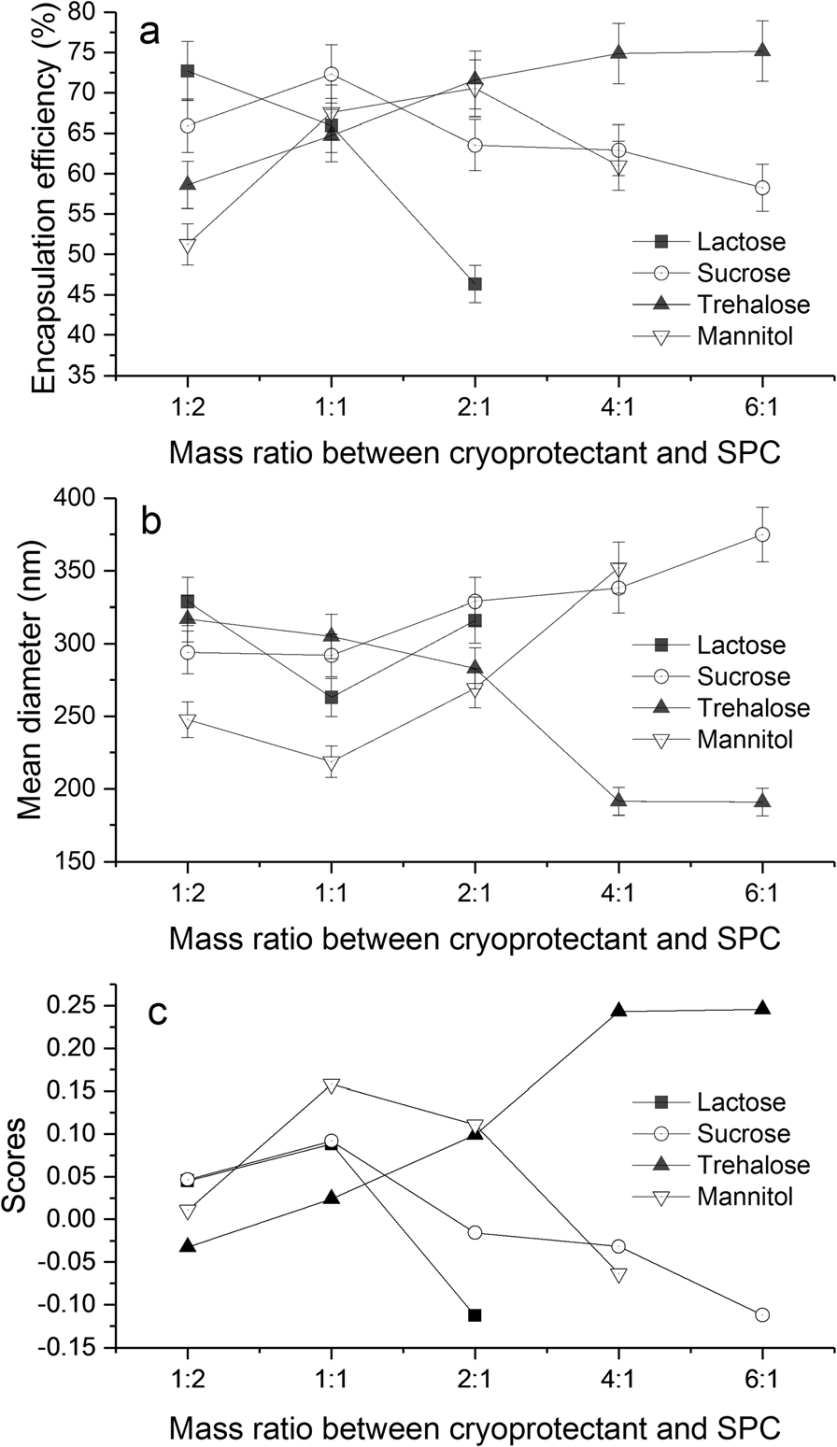
The effect of mass ratio between cryoprotectant and SPC on encapsulation efficiency (**a**), mean diameter (**b**) and scores (**c**) of reconstituted liposomes (mean  ±  SD, *n* = 3)

Competition for liquid water between the growing ice crystals and the hydrophilic substances (including the hydrophilic portion of the lipid membrane) during freezing leads to adhesion of ice crystals to the phospholipid groups. This can result in damage to the lipid membrane. Lipid membrane fusion following rehydration causes an increase in particle size and leakage of encapsulated drug. Lyoprotectant can reduce liposomal damage during the freeze-thaw process [32]. In this study, we investigated the effect of various types (lactose, sucrose, trehalose, mannitol) and dosage (lyoprotectant to SPC ratio was 1:2, 1:1, 2:1, 4:1, and 6:1 *w*/*w*) of lyoprotectant on scores of reconstituted liposome. Single-factor experiments were performed while maintaining all other variables constant: GA amount of 60 mg, SPC amount of 508 mg, cholesterol amount of 151 mg, volume percentage of TBA in the co-solvent of 55%, pre-freeze temperature of − 40 °C, pre-freeze time of 12 h. Experimental results are shown in Fig. 9. The encapsulation efficiency increases firstly and then decreases by decreasing lyoprotectant/SPC weight ratio from 1:2 to 1:6, wherein lactose, sucrose, and mannitol are respectively used as lyoprotectant. However, the encapsulation efficiency of the trehalose group increases constantly with decreasing lyoprotectant/SPC weight ratio (Fig. 9a). In terms of the mean diameter (Fig. 9b), it was found that the mean diameter was greater than 218 nm for lactose, sucrose, and mannitol group in the range from 1:2 to1:6. Nevertheless, the mean diameter of trehalose group can be reduced to less than 190 nm when lyoprotectant/SPC weight ratio is more than 4:1; obviously, the protective effect of trehalose is better than other lyoprotectants tested. Trehalose has a good protection ability for membrane, perhaps because of the formation of hydrogen bonds with the polar head groups of lipids, and disruption of the tetrahedral hydrogen bond network of water [33]. According to scores (Fig. 9c), the highest score (0.24) was obtained when trehalose/SPC weight ratio is 4:1 and 6:1. Finally, we choose trehalose and 4:1 (trehalose/SPC weight ratio) for following experiments from the perspective of cost and increasing drug loading.

Through the above Box-Benhnken design and lyoprotectant screening experiment, the experimental conditions were determinated: GA amount of 60 mg, SPC amount of 508 mg, cholesterol amount of 151 mg, volume percentage of TBA in the co-solvent of 55%, weight ratio of trehalose to SPC was 4:1. Under these conditions, the encapsulation efficiency and mean diameter were 74.87% and 191 nm, respectively.

### Transmission Electron Microscopy

**Fig. 10:**
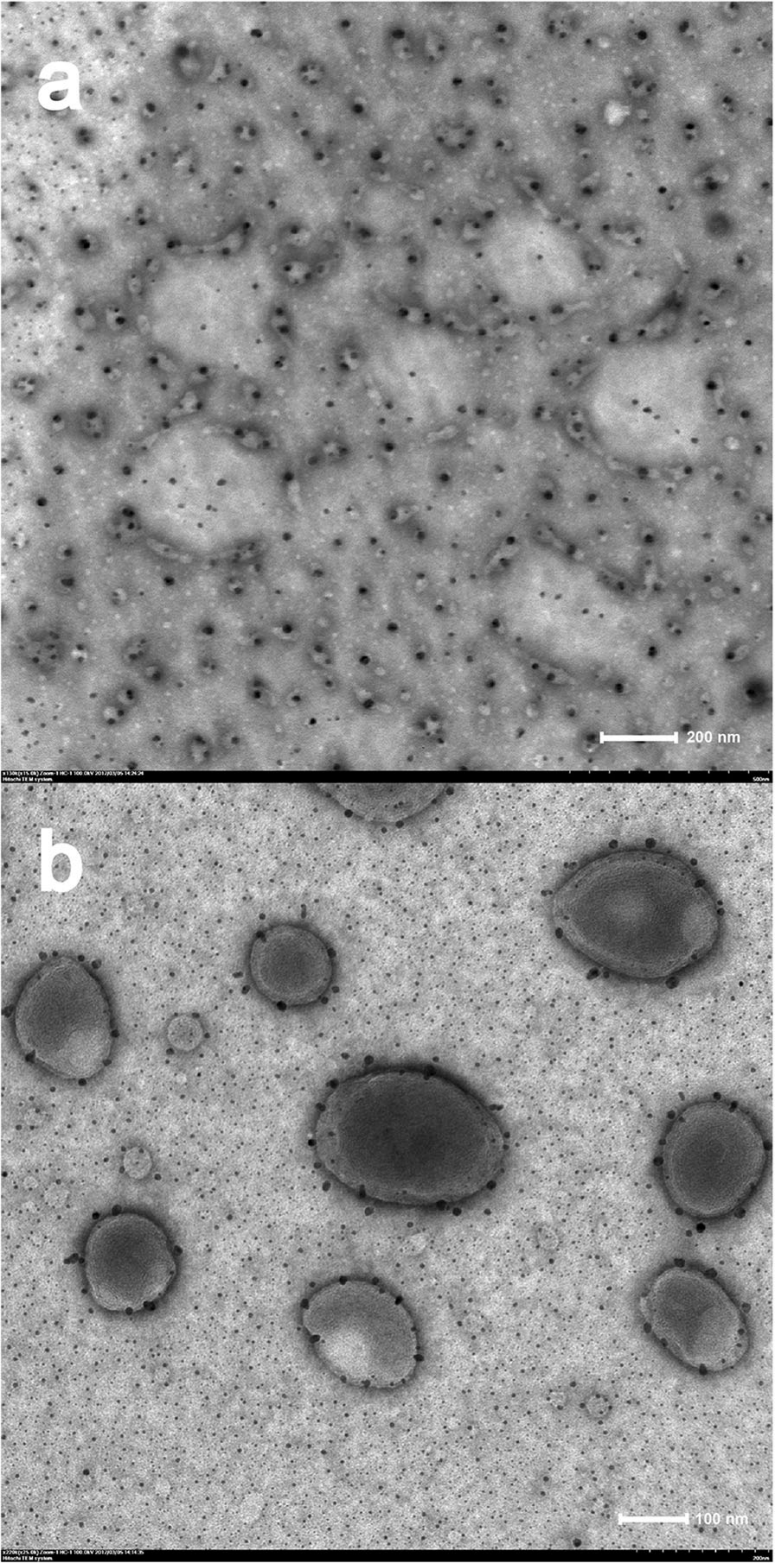
Transmission electron micrographs of reconstituted liposomes, (**a**) initial state of hydration of proliposomes, (**b**) fully assembled liposomes

In this study, TEM of liposomes suspension was taken at the same time point (same hydration time). We have observed different states in the sample, which could explain the self-assembly behavior of the liposomes. Figure 10a shows the initial state of hydration; it can be seen that a large amount of GA (black dots) is wrapped in dispersed phospholipids (translucent material), and spontaneous aggregation of the phospholipid fragments occurs. Figure 10b shows the morphology of fully assembled liposomes (average diameter of about 200 nm), which were nearly spherical with a phospholipid bilayer structure (the light-gray portion). Moreover, the drug particles (dark gray dots) were entrapped in the lipid bilayer.

### Stability of GA Proliposome

After 6 months, the proliposome powders have a good mobility and an unaltered appearance. The liposome suspension formed automatically when in contact with purified water. The entrapment efficiency and particle size of the reconstituted liposome were 72.82% and 198 nm. There is no significant difference from the data of the reconstituted liposome 6 months before. Therefore, the GA proliposome could be considered stable at 25 °C for over 6 months.

### In Vitro Drug Release Studies

**Fig. 11:**
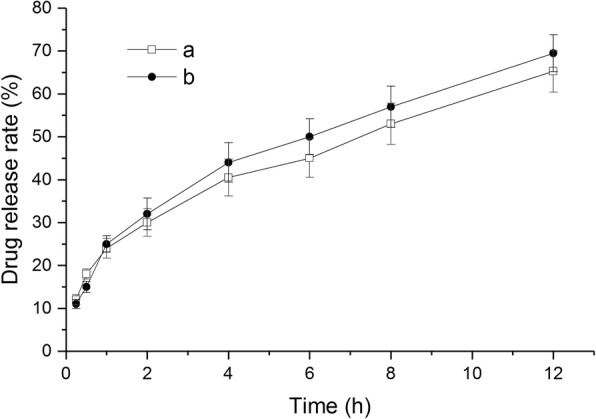
In vitro dissolution profiles of GA from GA-loaded liposomes in a PBS and b physiological saline solution (mean ± SD, *n* = 3)

Evaluation of in vitro drug release from encapsulated liposome was done by dialysis method. The in vitro release profiles of GA from GA-loaded liposomes at 37 °C in PBS (pH 7.4) and physiological saline solution are shown in Fig. 11. The release profile of both group showed a fast release (the larger slope) within 1 h, then curve slope becomes smaller after 1 h, the release rate begins to slow down. The drug-release curve shapes of physiological saline solution group are similar to PBS group. The in vitro release of GA from the GA-loaded liposomes was 65.25 ± 4.82% and 69.46 ± 4.32% from PBS and physiological saline solution in 12 h. No significant difference (*P* = 0.088, paired *t* test, SPSS software17.0) was found for the release of GA at different release medium over the entire study period, which demonstrated that the reconstituted liposomes have both sustained-release performance in two kinds of release medium.

### In Vitro Cell Uptake

**Fig. 12:**
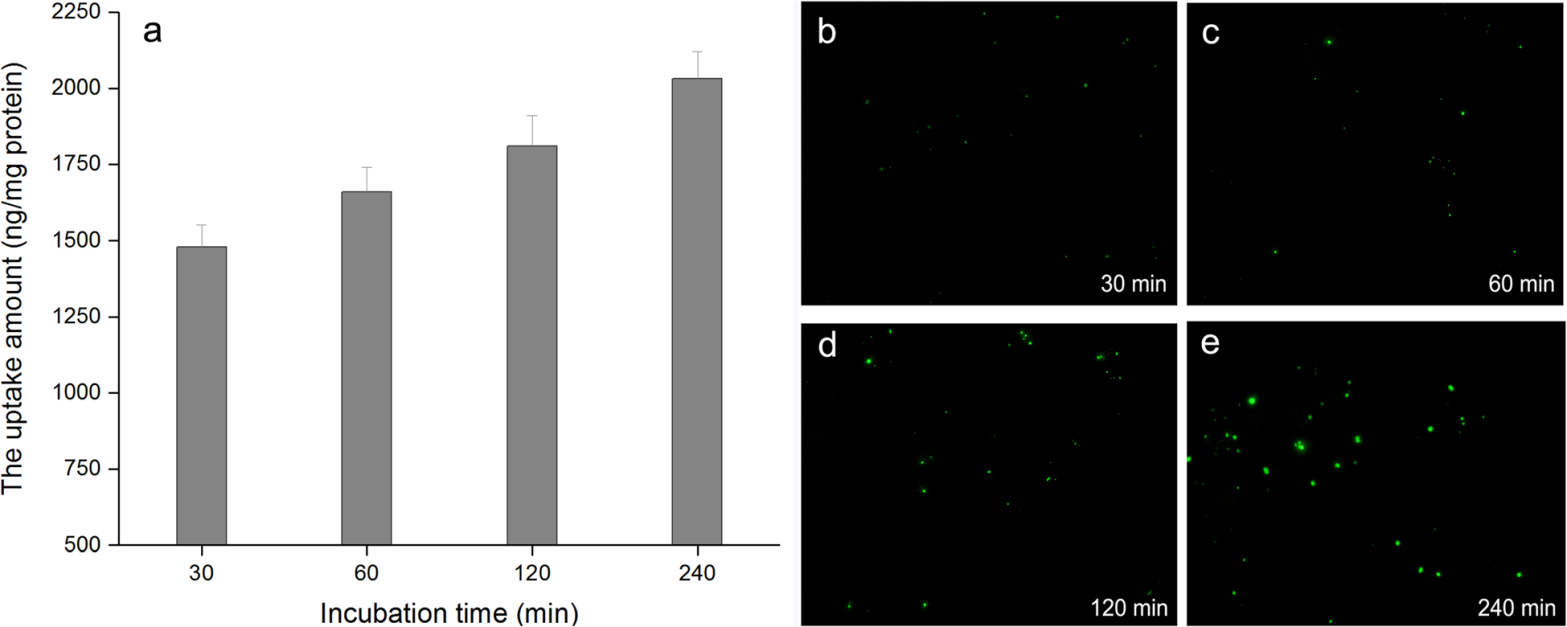
In vitro cellular uptake of GA-loaded liposomes by Hep G2 cells. **a** The uptake amount versus incubation time. **b**–**e** Fluorescence microscopy images at 30, 60, 120, and 240 min

Figure 12a showed that the uptake process of GA-liposomes by Hep G2 cells is time-dependent under the experimental concentrations. After incubation for 30 min, the uptake amounts of drug-loaded liposomes (unit mass protein) by Hep G2 cells were 1480 ng. In the range from 30 to 240 min, the uptake amounts of drug-loaded liposomes (unit mass protein) were gradually increased from 1480 to 2030 ng. Figure 12b–e showed fluorescence microscopy images of Hep G2 cells at 30, 60, 120, and 240 min after ingestion of drug-loaded liposomes, and it is observed that the fluorescence intensity is also gradually increased over time. This result indicates that the reconstituted liposomes prepared by monophase solution method can be effectively uptaken by the hepatoma cells.

## Conclusions

In the present work, preformulation investigation, formulation design along with in vitro characterization of GA-loaded liposomes by lyophilization monophase solution method have been done. After carrying out a preformulation study, we found that solubility of GA, cholesterol, and SPC in TBA/water co-solvent was substantially increased when temperature was over 40 °C. Sublimation rate of co-solvent gradually increased with increasing TBA volume percentage, which perhaps relate to surface morphology of the frozen co-solvent and saturated vapor pressure. After lyophilization using TBA/water co-solvent system, GA became amorphous structure; moreover, water solubility increased. This may have an effect on proliposome encapsulation during hydrated reconstruction. After optimization by Box-Benhnken design and screening of lyoprotectant, the optimum conditions (508 mg SPC, 151 mg cholesterol, 55% volume percentage of TBA, 4:1 trehalose/SPC weight ratio) for lyophilization monophase solution process were achieved. Under the optimum conditions, satisfactory encapsulation efficiency (74.87%) and mean diameter (191 nm) of reconstituted liposomes were obtained. The reconstituted liposomes resulted in initial assemble and final spherical shape, as confirmed by TEM analysis. The in vitro release profile of the produced GA-loaded liposome was investigated in the two media and it both showed prolonged release during 12 h. Cellular uptake studies showed that the uptake process of reconstituted liposomes by Hep G2 cells is time-dependent.

## Abbreviations

BBD: Box-Benhnken design
DSC: Differential scanning calorimetry
EE: Encapsulation efficiency
FTIR: Fourier transform infrared spectroscopy
GA: Glycyrrhetinic acid
MD: Mean diameter
SPC: Soybean phosphatidylcholine
TBA: Tert-butyl alcohol
TEM: Transmission electron microscopy
XRD: X-ray diffraction
